# CDK2 Regulates HIV-1 Transcription by Phosphorylation of CDK9 on Serine 90

**DOI:** 10.1186/1742-4690-9-94

**Published:** 2012-11-09

**Authors:** Denitra Breuer, Alexander Kotelkin, Tatiana Ammosova, Namita Kumari, Andrey Ivanov, Andrey V Ilatovskiy, Monique Beullens, Philip R Roane, Mathieu Bollen, Michael G Petukhov, Fatah Kashanchi, Sergei Nekhai

**Affiliations:** 1 Center for Sickle Cell Disease, Department of Medicine, Howard University, 1840 7th Street, N.W. HURB1, Suite 202, Washington, DC, 20001, USA; 2 Department of Microbiology, Howard University, Washington, DC, 20059, USA; 3 Division of Molecular and Radiation Biophysics, Petersburg Nuclear Physics Institute, Gatchina, Russia; 4 Research and Education Center “Biophysics”, PNPI RAS and St. Petersburg State Polytechnical University, St. Petersburg, Russia; 5 Department of Cellular and Molecular Medicine, University of Leuven, Leuven, Belgium; 6 National Center for Biodefense and Infectious Diseases, George Mason University, Manassas, VA, 20110, USA

## Abstract

**Background:**

HIV-1 transcription is activated by the viral Tat protein that recruits host positive transcription elongation factor-b (P-TEFb) containing CDK9/cyclin T1 to the HIV-1 promoter. P-TEFb in the cells exists as a lower molecular weight CDK9/cyclin T1 dimer and a high molecular weight complex of 7SK RNA, CDK9/cyclin T1, HEXIM1 dimer and several additional proteins. Our previous studies implicated CDK2 in HIV-1 transcription regulation. We also found that inhibition of CDK2 by iron chelators leads to the inhibition of CDK9 activity, suggesting a functional link between CDK2 and CDK9. Here, we investigate whether CDK2 phosphorylates CDK9 and regulates its activity.

**Results:**

The siRNA-mediated knockdown of CDK2 inhibited CDK9 kinase activity and reduced CDK9 phosphorylation. Stable shRNA-mediated CDK2 knockdown inhibited HIV-1 transcription, but also increased the overall level of 7SK RNA. CDK9 contains a motif (^90^SPYNR^94^) that is consensus CDK2 phosphorylation site. CDK9 was phosphorylated on Ser90 by CDK2 *in vitro*. In cultured cells, CDK9 phosphorylation was reduced when Ser90 was mutated to an Ala. Phosphorylation of CDK9 on Ser90 was also detected with phospho-specific antibodies and it was reduced after the knockdown of CDK2. CDK9 expression decreased in the large complex for the CDK9-S90A mutant and was correlated with a reduced activity and an inhibition of HIV-1 transcription. In contrast, the CDK9-S90D mutant showed a slight decrease in CDK9 expression in both the large and small complexes but induced Tat-dependent HIV-1 transcription. Molecular modeling showed that Ser 90 of CDK9 is located on a flexible loop exposed to solvent, suggesting its availability for phosphorylation.

**Conclusion:**

Our data indicate that CDK2 phosphorylates CDK9 on Ser 90 and thereby contributes to HIV-1 transcription. The phosphorylation of Ser90 by CDK2 represents a novel mechanism of HIV-1 regulated transcription and provides a new strategy for activation of latent HIV-1 provirus.

## Background

Despite efficient anti-retroviral therapy, eradication of HIV-1 infection is challenging and requires novel biological insights and therapeutic strategies. Eradication of latent HIV-1 provirus is especially challenging as integrated HIV-1 is not affected by the existing anti-HIV-1 drugs until its transcription is activated [1]. HIV-1 transcription from HIV-1 LTR depends on both host cell factors and the HIV-1 transactivation Tat protein [2]. While latency is mostly studied on HIV-1 subtype B, other HIV-1 subtypes have different configuration of HIV-1 LTR that may determine transcription activity of the integrated provirus [3]. In a model of acute HIV-1 infection, HIV-1 subtypes A, B, C, D, F and AG had similar latency profiles while subtype AE showed a reduced potential for latency, which correlated with the presence of GABP instead of NF-κB transcription factor binding site in the LTR [3]. Major function of HIV-1 Tat protein is to recruit the positive transcription elongation factor b (P-TEFb), that contains the cell cycle-dependent kinase (CDK) 9 and cyclin T1 to the TAR RNA, a hairpin-loop structure located at the 5^′^-end of all nascent HIV-1 transcripts [4]. The HIV-1 TATA box and its flanking regions are recognized by the cellular pre-initiation complexes that require CTGC motifs for the accurate formation, which is disrupted by TAR RNA that may contribute to the establishment of latency [5]. Nucleosomal structure of integrated HIV-1 provirus may also contribute to the establishment of HIV-1 latency. HIV-1 chromatin-associated Spt6 maintains chromatin structure in the wake of RNAPII and is protected by Proteasomal ATPase-associated factor 1 (PAAF1) from proteasomal degradation [6]. Knockdown of either Spt6 or PAAF1 leads to the loss of histones from HIV-1 genomic DNA and production of transcripts defective for protein synthesis [6]. P-TEFb in the cells exists in two molecular weight forms. The lower molecular weight kinase active form of P-TEFb consists of CDK9 and cyclin T1 [7,8]. The high molecular weight inactive form of P-TEFb contains 7SK RNA, a dimer of CDK9/cyclin T1 and several additional proteins including HEXIM1 dimer, La-related LARP7 protein [9-11] and the methylphosphatase capping enzyme MePCE [12,13]. HEXIM1 binds with its inhibitory PYNT sequence to the active site of CDK9 [14]. The high molecular weight complex plays an important role in the activation of HIV-1 transcription, as it serves as a source of P-TEFb for the recruitment by HIV-1 Tat [15]. The importance of the CDK9 component of the large form of P-TEFb has been demonstrated by the effect of the CDK9 inhibitors, flavopiridol and DRB, which reduce the amount of the large form of P-TEFb and inhibit HIV-1 replication [16]. HIV-1 Tat protein was recently shown to recruit P-TEFb to HIV-1 pre-initiation complex in inactive form along with 7SK RNA and that formation of TAR RNA can displace 7SK RNA [17]. Tat was also shown to facilitate the formation of super-elongation complex (SEC) containing active P-TEFb and additional elongation factors and co-activators [18,19]. P-TEFb triggers elongation of RNA polymerase II (RNAPII) transcription by phosphorylating the negative elongation factor (NELF) and the DRB-sensitivity inducing complex (DSIF/Spt4/Spt5), which promotes the release of NELF [20]. P-TEFb also phosphorylates the Ser-2 residues of the C-terminal domain (CTD) of the largest subunit of RNA Polymerase II (RNAPII). Our previous studies also implicated CDK2 in the regulation of HIV-1 transcription [21,22]. The knock-down of CDK2 inhibited HIV-1 transcription and replication [23]. CDK2 was found to associate with the HIV-1 promoter *in vitro*[21] and *in vivo*[24]. Inhibition of CDK with small molecule inhibitor roscovitin [25], inhibited HIV-1 replication and prevented the association of CDK2 with the HIV-1 promoter [24]. Roscovitin and its analog CR8, also inhibited CDK9 activity [16], suggesting that CDK2 and CDK9 might be functionally linked [26,27]. Further support for this functional link came from the observation that inhibition of CDK2 by iron chelators inhibited CDK9 activity and HIV-1 transcription [28,29]. CDK9 has at least ten phosphorylation sites. Phosphorylation of Thr186 is critical for the activity of CDK9 [30,31] and the association of CDK9/cyclin T1 with 7SK RNA snRNP [30,31]. Autophosphorylation of Thr29 [32] and the C-terminal residues Ser329, Thr330, Thr333, Ser334, Ser347, Thr350, Ser353, and Thr354 [30] has also been reported. We recently showed that dephosphorylation of the T-loop Ser 175 residue by protein phosphatase-1 induces CDK9 activity and activates HIV-1 transcription [33].

Here, we investigated the phosphorylation of CDK9 by CDK2, using a siRNA and shRNA-mediated knockdown approach. We identified Ser 90 of CDK9 as a novel and key CDK2 phosphorylation site, and analyzed its effect on the distribution of P-TEFb between large and small molecular weight complexes and the ability of CDK9 to activate HIV-1 transcription. We also modeled Ser90 in the CDK9 structure.

## Results

### The transient knockdown of CDK2 by siRNA inhibits CDK9 activity

**Figure 1:**
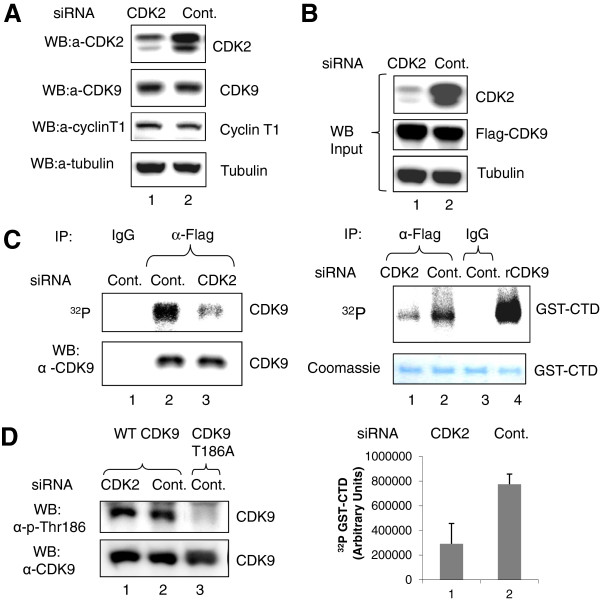
**Inhibition of CDK9 activity and phosphorylation by CDK2-directed siRNA. (A-B)** Inhibition of CDK2 expression and CDK9 activity. Lysates from 293T cells transfected with CDK2-directed (lane 1) or control siRNA (lane 2) were analyzed by immunoblotting for the expression of CDK2, CDK9, cyclin T1 and tubulin (panel A)Panel B, the cells were also co-transfected with Flag-CDK9 and cyclin T1 expression vectors. Lysates were immunoblotted with antibodies against CDK2, Flag and α-tubulin as loading control (upper panel) or immunoprecipitated with anti-Flag antibodies (lanes 1 and 2) or non-specific IgGs (lane 3) (lower panel). CDK9 activity was analyzed with GST-CTD as substrate. Lane 4, control recombinant CDK9/cyclin T1. GST-CTD is shown as Coomassie stain. Quantification is shown as average from three independent experiments. **(C)** Inhibition of CDK9 phosphorylation. Lysates from 293T cells transfected with CDK2-directed (lane 3) or control siRNA (lanes 1 and 2) and also with a vector expressing Flag-CDK9 WT as in panel B then pulse-labeled with (^32^P) were immunoprecipitated with anti-Flag antibodies (lanes 2 and 3) or with non-specific IgGs (lane 1), resolved on 10% SDS PAGE and exposed to a Phosphor imaging device (upper panel) or analyzed by immunoblotting with anti-CDK9 antibodies (lower panel). **(D)** No effect on CDK9 Thr186 phosphorylation. Lysates from 293T cells transfected CDK2-directed (lane 1) or control siRNA (lanes 2 and 3) and then co-transfected with vectors expressing Flag-CDK9 WT (lanes 1 and 2) or Flag-CDK9 T186A (lane 3) were immunoprecipitated with anti-Flag antibodies and analyzed by immunoblotting with Thr186 phospho-specific or anti-CDK9 antibodies.

Previously, we utilized siRNA-mediated CDK2 knockdown (KD) to demonstrate the importance of CDK2 for HIV-1 transcription [23]. Here, we analyzed the effect of CDK2 KD on CDK9 activity and phosphorylation. 293T cells were transiently transfected with CDK2-targeted siRNA or a control siRNA that resulted in the inhibition of CDK2 expression but had no effect on the expression of endogenous CDK9 and cyclin T1 or tubulin (Figure 1A, lanes 1 and 2). To measure CDK9 activity, the siRNA-transfected cells were co-transfected with Flag-tagged CDK9 and cyclin T1, and CDK9 was immunoprecipitated with anti-Flag antibodies. Expression of Flag- CDK9 was not affected by CDK2 KD (Figure 1B, upper panel). CDK9 activity, analyzed with GST-CTD as a substrate, was reduced in the cells transfected with CDK2-targeted siRNA but not in the cells transfected with control siRNA (Figure 1B, middle panel, lanes 1 and 2). These results suggest that the transient inhibition of CDK2 has a negative effect on CDK9 activity.

### The loss of CDK2 inhibits CDK9 phosphorylation

Because CDK9 kinase activity is regulated by phosphorylation [30-32], we analyzed the overall level of CDK9 phosphorylation in the cells transfected with CDK2-directed siRNA. The siRNA-transfected 293T cells were re-transfected with Flag-tagged CDK9 and then treated with (^32^P) orthophosphate to allow CDK9 phosphorylation. CDK9 was precipitated with anti-Flag antibodies from the cellular lysates, and CDK9 phosphorylation was analyzed by Phosphor Imaging. CDK9 phosphorylation was reduced in the cells transfected with CDK2-targeted siRNA in comparison to the control siRNA (Figure 1C, lanes 2 and 3) suggesting that the inhibition of CDK2 reduces the overall level of CDK9 phosphorylation. Because CDK9 activity is regulated by Thr186 phosphorylation [30,31], we analyzed Thr186 phosphorylation in the cells transfected with CDK2-directed siRNA. Flag-tagged WT CDK9 and CDK9 T186A mutant were co-transfected into the siRNA transfected cells. CDK9 was precipitated with anti-FLAG antibodies, and Thr186 phosphorylation was analyzed with phospho-specific antibodies. Phosphorylation of Thr186 was similar in the cells transfected with CDK2-directed siRNA and the cells transfected with control siRNA (Figure 1D, lanes 1 and 2), suggesting that CDK2 knock-down has no effect on Thr186 phosphorylation. The specificity of the antibodies was confirmed with the CDK9 T186A mutant, which showed no Thr186 phosphorylation (Figure 1D, lane 3). These results suggest that CDK KD has a negative effect on overall CDK9 phosphorylation which was not related to Thr186 phosphorylation.

### The stable knockdown of CDK2 inhibits HIV-1 Transcription

**Figure 2:**
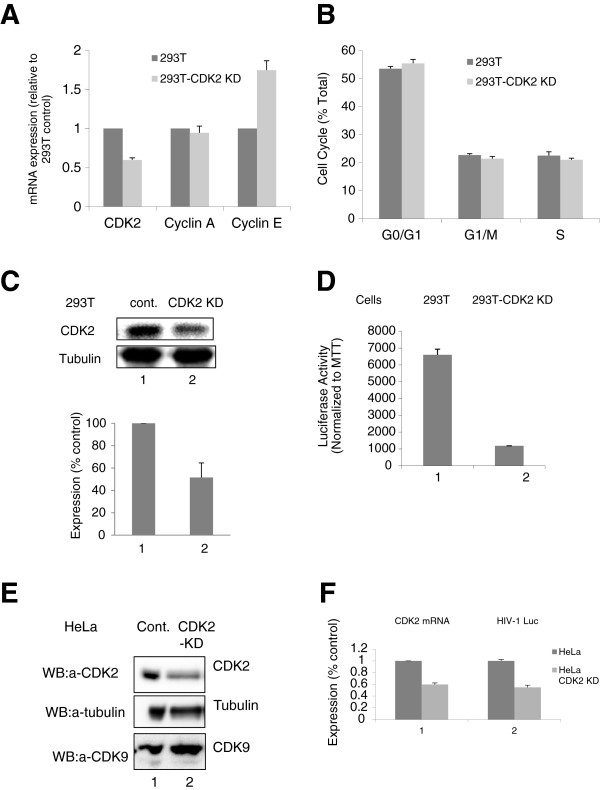
**CDK2 KD inhibits HIV-1 transcription. (A**) Expression of CDK2 mRNA is reduced in CDK2 KD cells. Total RNA was extracted from 293T cells and 293T-CDK KD cells lysates, reverse transcribed and analyzed by real-time PCR. β-Actin was used as an internal control. Quantification is shown in triplicates. **(B)** Cell cycle analysis of 293T-59 cells. Cells were fixed with 70% ethanol, stained with Propidium Iodine and analyze by FACS. Quantification is shown in triplicates. **(C)** CDK2 protein expression is reduced in 293T-CDK KD cells. Lysates from 293T and 293T-CDK2 KD cells (lanes 1 and 2) were analyzed for CDK2 expression by western blotting. Quantification is shown for three independent experiments. **(D)** Inhibition of VSVG HIV-1 Luc replication. 293T and 293T-CDK2-KD cells were infected with VSVG-pNL4-3 Luc virus and luciferase activity was measured. MTT assay was used for the normalization. Quantification is shown for three independent experiments. **(E)** CDK2 protein expression is reduced in HeLa-CDK2 KD cells. CDK2, tubulin and CDK9 expression was analyzed in HeLa and HeLa-CDK2 KD cells (lanes 1 and 2) by western blotting. **(D)** Inhibition of VSVG HIV-1 Luc replication. Lane1, CDK2 expression was analyzed in HeLa and HeLa-CDK2 KD cells by Real-time PCR. 18S RNA was used as an internal control. Quantification is shown in triplicates. Lane 2, Luciferase expression of VSVG-HIV-1 Luc was analyzed in infected HeLa and HeLa -CDK2-KD cells at 48 hours post infection. MTT assay was performed for the normalization. Quantification is shown for three independent experiments.

To further analyze the effect of CDK2 inhibition on HIV-1 transcription, we first generated stable cell lines that express CDK2-targeting shRNAs using vectors that target several distinct sequences of CDK2 (see Experimental Procedures). Only 293T cells expressing the shRNA that targeted the ^1010^atggacggagcttgttatc^1028^ sequence (OS211959) showed about 40% reduction of CDK2 mRNA (Figure 2A, CDK2; 293T-CDK2 KD). In contrast, no significant change was observed for cyclin A mRNA expression (Figure 2A). Interestingly, cyclin E mRNA expression level was increased in 293T-CDK2 KD cells (Figure 2A). Cell cycle analysis of the 293T-CDK2 KD cells by FACS showed no significant difference in comparison to the parental 293T cells (Figure 2B), suggesting that a partial knockdown of CDK2 has no significant effect on the cell cycle progression and is not cytotoxic. Analysis of protein expression showed about 40% reduction in CDK2 expression in 293T-CDK2 KD cells (Figure 2C, lane 2).

To analyze the effect of stable CDK2 knock-down on HIV-1 transcription, 293T-CDK2 KD cells were infected with VSVG-pseudotyped pNL-4-3 Luc, which expresses luciferase in place of *nef*. For normalization, MTT assay was used. Luciferase activity was significantly reduced in 293T-CDK2 KD cells compared to 293T cells (Figure 2D), suggesting that reduction of CDK2 expression suppresses HIV-1 transcription. Similar results were obtained when 293T-CDK KD cells were co transfected with an HIV-1 genomic clone, pNL-4-3 Luc that expresses luciferase in place of nef and CMV-*Lac Z* vector which showed reduction of luciferase normalized to β-galactosidase activity in 293T-CDK2 KD cells compared to 293T cells (data not shown). We also generated HeLa CDK2 KD cell line, which showed reduced CDK2 protein expression (Figure 2E, lane 2) and CDK2 mRNA expression (Figure 2F, lane 1). Infection with VSVG-HIV-1 Luc also showed reduced luciferase expression in comparison to parental HeLa cells (Figure 2F, lane 2). Together, these results indicate that stable CDK2 KD inhibits HIV-1 transcription and single round HIV-1 replication.

### Stable CDK2 KD increases 7SK RNA levels

**Figure 3:**
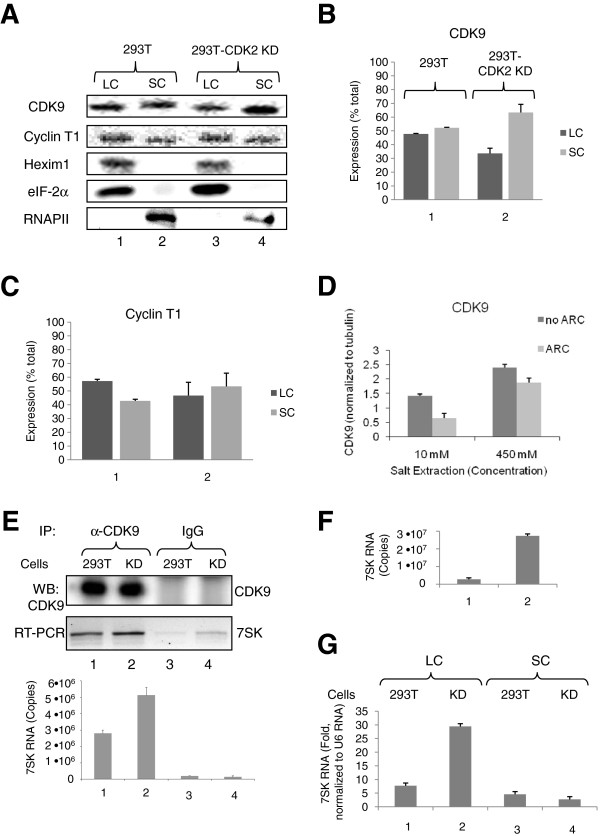
**Stable expression of CDK2-directed shRNA induces 7SK RNA expression. (A-C)** Effect of CDK2 KD on small and large P-TEFb complexes. Lysates from 293T and 293T-CDK2 KD cells sequentially extracted with low and high salt buffers, were analyzed by immunoblotting for CDK9, cyclin T1, Hexim 1, eIF-2α and RNAPII. Panels B and C show average results from three experiments. **(D**) CDK9 inhibitor ARC prevents CDK9 with association with large P-TEFb complex. Lysates from 293T cells untreated or treated with 10 μM ARC and extracted with low salt buffer (10 mM) or high salt buffer (450 mM) were resolved on 10% SDS-PAGE and analyzed by immunoblotting for CDK9 and tubulin. Average CDK9 expression adjusted to tubulin from two separate experiments is shown. **(E)** CDK2 KD increases the amount of 7SK RNA associated with CDK9. RNA isolated from co-immunoprecipitates with anti-CDK9 antibodies or control IgGs from 239T or 293T-CDK2 KD whole cell lysates was reverse transcribed and analyzed by semi-quantitative (30 cycles, upper panel) or real-time PCR (lower panel). Results are presented as numbers of copies of 7SK RNA. **(F)** CDK2 KD increases total 7SK RNA amount. 7SK RNA was analyzed in whole cell lysates of 293T or 293T-CDK2 KD cells by real-time PCR using 7SK expression vector as control. Results are presented as numbers of copies of 7SK RNA. **(G)** CDK2 KD increases 7SK RNA in the large complex fraction. 7SK RNA was analyzed by real-time PCR using U6 RNA as reference. Quantification is shown in triplicates.

To analyze the expression of P-TEFb in the large and small complexes of 293T-CDK2 KD cells, we initially followed the protocol developed by David Price and his colleagues [16] in which the large P-TEFb complex was extracted during cell lysis with a buffer containing low salt concentration, and the small complex was recovered with subsequent extractions using a high-salt buffer. Large and small complexes were extracted from 293T and 293T-CDK2 KD cells. The low salt extraction contained cytoplasmic proteins as evidenced by the presence of eIF-2α protein (Figure 3A, lanes 1 and 3), whereas nuclear proteins were extracted with high salt as evidenced by the presence of RNAPII (Figure 3A, lanes 2 and 4). HEXIM1 was extracted with low salt suggesting the extraction of large P-TEFb complex (Figure 3A, lanes 1 and 3). There was less CDK9 in the large complex and more in small complex present in 293T-CDK2 KD cells in comparison to the 293T cells (Figure 3A and B). Correspondingly, there was more cyclin T1 present in the small complex in 293T-CDK2 KD cells (Figure 3C). To further confirm that low salt extraction reflected large P-TEFb complex, we analyzed the effect of CDK9 inhibitor, ARC, on the presence of CDK9 in the large complex because CDK9 inhibitors were shown to reduce CDK9 association with the large P-TEFb complex [16]. Treatment with 10 μM ARC significantly reduced CDK9 in low salt but not in the high salt extracts (Figure 3D), further confirming that low salt extract contains large P-TEFb complex. To further investigate the association of CDK9 with the large P-TEFb complex, we determined the amount of 7SK RNA associated with P-TEFb in CDK2 KD cells. CDK9 was immunoprecipitated from 293T and 293T-CDK2 KD cells lysates and the associated 7SK RNA was analyzed by RT-PCR. A semiquantitative and quantitative RT-PCR showed, unexpectedly, more 7SK RNA associated with P-TEFb in 293T-CDK2 KD cells compared to 293T cells (Figure 3E, upper and low panels correspondently). However, total 7SK RNA levels were much higher in 293T KD cells (Figure 3F, lane 2). When we analyzed 7SK RNA in the low salt and high salt lysates, much higher levels of 7SK RNA were found in the low salt extracts from 293T-CDK2 KD cells compared to 293T cells (Figure 3G, lanes 1 and 2). Smaller amounts of 7SK RNA were found in the high slat lysates of 293T and 293T-CDK2 KD cells (Figure 3G, lanes 3 and 4). Thus, because of this unexpected increase in the total amount of 7SK RNA in 293T-CDK2 KD cells, we could not conclude with definition whether CDK9 phosphorylation has a direct effect on the association with the large P-TEFb complex.

### CDK2 phosphorylates Serine 90 of CDK9

**Figure 4:**
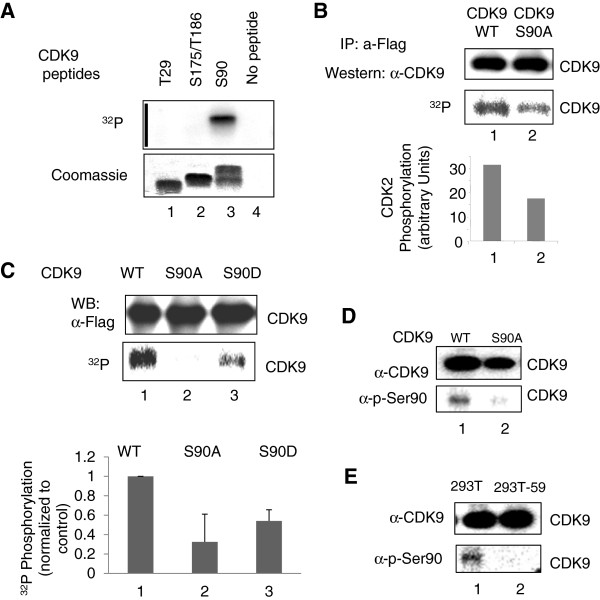
**CDK2 phosphorylates Ser90 residue of CDK9. (A)** CDK2 phosphorylates Ser90-containing peptide. CDK9-derived chemically synthesized peptides containing Thr29; Ser175 and Thr186; or Ser90 residues were phosphorylated by recombinant CDK2/cyclin E and analyzed by Phosphor Imaging Device (upper panel) or stained with Coomassie (lower panel). **(B)** CDK9 S90A mutant is less phosphorylated by CDK2/cyclin E *in vitro*. WT CDK9 and CDK9 S90A were expressed in 293T cells, precipitated with anti-FLAG antibodies and incubated with recombinant CDK2/cyclin E in the presence of (^32^P) ATP. The reactions were resolved on a 10% SDS Tris-glycine gel and analyzed by immunoblotting (upper panel) or on Phosphor Imaging Device (lower panel). Quantification from the Phosphor Imager is shown. **(C)** Substitution of CDK9 Ser 90 prevents its phosphorylation in cultured cells. 293T cells transfected with FLAG-tagged WT CDK9, CDK9 S90A and CDK9 S90D and also co-transfected with Cyclin T1, pulse-labeled with (^32^P) and CDK9 was immunoprecipitated from cellular lysates with anti-FLAG antibodies, and analyzed by immunoblotting (upper panel) or by Phosphor Imaging Device (lower panel). Quantification of the bands on Phosphor Imager is shown for three independent experiments. **(D and E)** CDK9 Ser90 phosphorylation is decreased in CDK2 KD cells. 293T cells were transfected with vectors expressing Flag-CDK9 WT or Flag-CDK9 S175A (panel D); or 293T and 293T-59 cells were transfected with a vector expressing Flag-CDK9 WT (panel E) and after 48 hrs in culture treated with 0.1 μM okadaic acid. CDK9 was immunoprecipitated with anti-Flag antibodies and analyzed by immunoblotting with anti-CDK9 or Ser90 phospho-specific antibodies.

A typical CDK2-phosphorylation site contains the (S/T)PX(K/R) sequence [34]. Analysis of the sequence of CDK9 revealed the presence of a S^90^PYNR^94^, which qualifies as a CDK2 consensus phosphorylation site. We analyzed CDK9 phosphorylation by CDK2 using peptides that span potential phosphorylation sites including N-terminal Thr29; the T-loop Ser175 and Thr186; and Ser90. CDK2 phosphorylated a peptide containing Ser90 (Figure 4A, lane 3) but not Thr29 or Ser175/Thr186 (Figure 4A, lanes 1 and 2).

We then analyzed whether CDK2 phosphorylates CDK9 Ser90 in the whole CDK9 protein *in vitro*. Flag-tagged WT CDK9 and CDK9 S90A mutants were expressed in 293T cells, immunoprecipitated with anti-FLAG antibodies and subjected to phosphorylation with recombinant CDK2/cyclin E. Phosphorylation of the CDK9 S90A mutant was reduced (Figure 4B, lane 2) in comparison to the phosphorylation of WT CDK9 (Figure 4B, lane 1), suggesting that CDK2 phosphorylates Ser90.

We next analyzed whether Ser90 can be phosphorylated in cultured cells. WT CDK9, CDK9 S90A and CDK9 S90D mutants were expressed in 293T cells. The cells were incubated in phosphate-free media and then treated with (^32^P) orthophosphate. CDK9 was immunoprecipitated with anti-FLAG antibodies and resolved on SDS-PAGE. CDK9 phosphorylation was analyzed with the Phosphor Imager. Both the S90A and S90D substitutions reduced CDK9 phosphorylation (Figure 4C, lanes 2 and 3), as compared to the phosphorylation of WT CDK9 (Figure 4C, lane 1), suggesting that CDK9 Ser90 is phosphorylated *in vivo*.

The phosphorylation of CDK9 on Ser90 was further investigated with Ser90 phospho-specific antibodies. The specificity of the antibodies was confirmed with the CDK9 S90A mutant, which showed no phosphorylation (Figure 4D compare lanes 1 and 2). The phosphorylation of CDK9 on Ser90 was decreased in the CDK2 KD cells (Figure 4E, compare lanes 1 and 2), suggesting that CDK2 KD affected Ser90 phosphorylation. Together, these results showed that CDK2 phosphorylates CDK9 on Ser90.

### Mutation of Ser90 alters the association of CDK9 with the large P-TEFb complex

**Figure 5:**
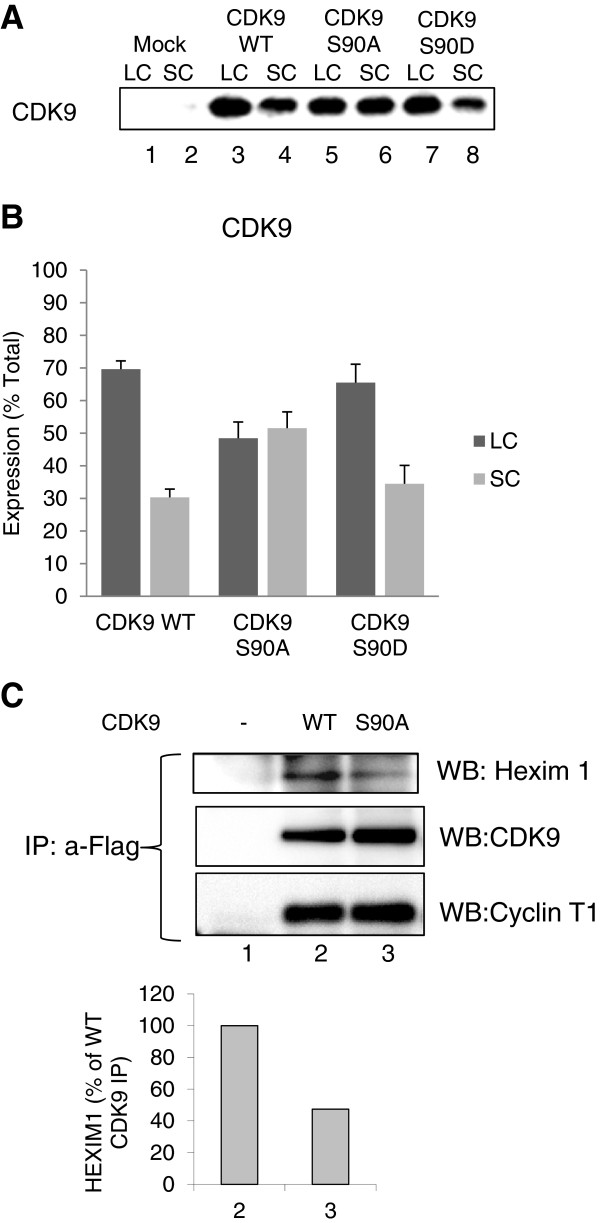
**Substitution of CDK9 serine 90 for alanine reduces CDK9 association with large P-TEFb complex.** 293T cells were transfected with Flag-tagged WT CDK9, CDK9 S90A and CDK9 S90D and also co-transfected with Cyclin T1-expressing vectors. At 48 hrs posttransfection, the cells were lysed with low salt (large complex – LC) buffer followed by high salt buffer (small complex – SC). CDK9 expression was analyzed by Western blotting. (**A)** Representative immunoblots. **(B)** Quantification of CDK9 in large (LC) and small (SC) complexes conducted in two independent experiments. **(C)** Co-immunoprecipitation analysis. 293T cells were transfected with Flag-tagged WT CDK9 and CDK9 S90A and also co-transfected with Cyclin T1 expressing vectors. At 48 hrs post-transfection, the cells were lysed; CDK9 was immunoprecipitated from cellular lysates with anti-Flag antibodies, resolved on 10% SDS PAGE and analyzed by immunoblotting with antibodies for Hexim1, CDK9 and cyclin T1. Lane 1, mock-transfected control.

To analyze the effect of Ser90 phosphorylation on the association of CDK9 and cyclin T1 with the large and small molecular weight complexes, 293T cells were transfected with WT CDK9, the non-phosphorylatable S90A mutant and the phosphorylation mimicking S90D mutant. The cells were also co-transfected with cyclin T1. Analysis of CDK9 expression in the large and small complexes showed a decrease of CDK9S90A in the large P-TEFb complex but not the WT CDK9 or CDK9 S90D (Figure 5A and B), suggesting that the CDK9 S90A mutant is less efficiently associated with the large P-TEFb complex. To further investigate the effect of CDK9 S90A mutant, we analyzed co-precipitation of CDK9, cyclin T1 and HEXIM1. CDK9 S90A mutant precipitated with about 50% less HEXIM1 while binding equal amount of cyclin T1 (Figure 5C, lane 3) suggesting that CDK9 S90A mutant less efficiently associated with the large P-TEFb complex.

### Ser90 of CDK9 affects HIV-1 transcription

**Figure 6:**
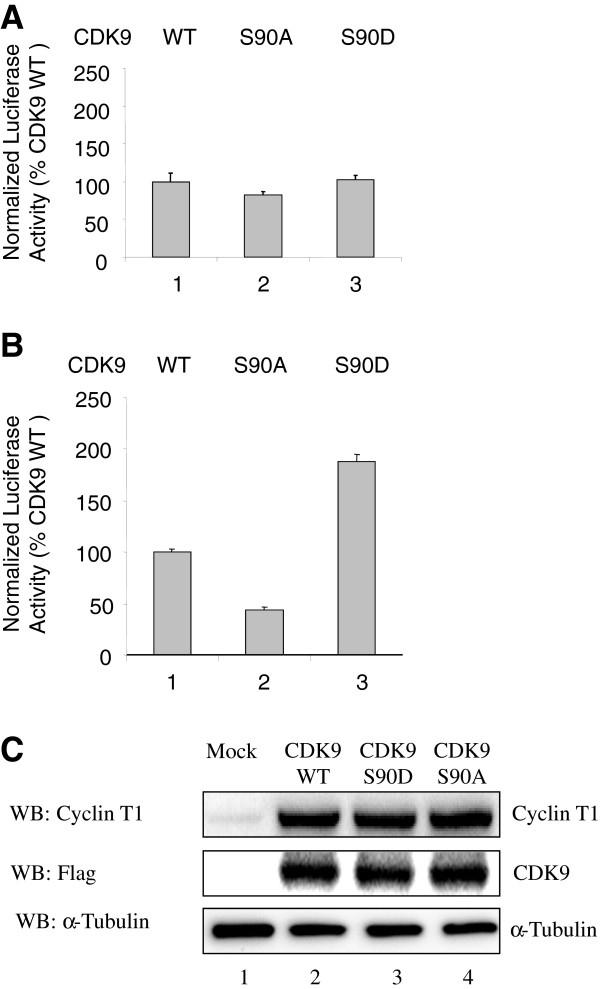
**CDK9 Ser 90 substitutions affect Tat-dependent HIV-1 transcription. (A-B)** 293T cells were transfected with HIV-1 LTR-Luc expression vector along with WT CDK9, CDK9 S90A and CDK9 S90D expression vectors without (panel A) or with Tat expression vector (panel B). Cells were lysed at 24 hours posttransfection and luciferase activity was measured followed by the measurement of EGFP fluorescence, which was used for the normalization. Quantification is shown for three independent experiments. **(C)** Expression of CDK9 and cyclin T1. To determine the levels of CDK9 and cyclin T1 expression, 293T cells were transfected with FLAG-tagged WT CDK9, CDK9 S90A and CDK9 S90D and also co-transfected with Cyclin T1 and HIV-1 Tat expression vectors. At 48 hrs post-transfection, the cells were lysed, the lysates were resolved on 10% SDS PAGE and analyzed by immunoblotting with antibodies for cyclin T1, CDK9, and tubulin was used as loading control. Lane 1, mock-transfected control.

We next analyzed the effect of Ser 90 mutations on HIV-1 transcription. The 293T cells were transfected with vectors expressing WT CDK9, CDK9 S90A and CDK9 S90D mutants and co-transfected with the HIV-1 LTR-Luc reporter and CMV-EGFP expressing vector (for normalization). In the absence of Tat, there was a small inhibitory effect of CDK9 S90A (about 20%), but no effect of CDK9 S90D on basal HIV-1 transcription (Figure 6A, lanes 1 to 3). In Tat-induced HIV-1 transcription (about 10-fold activation, not shown), the expression of CDK9 S90A inhibited HIV-1 transcription by about 60% whereas the expression of CDK S90D activated HIV-1 transcription nearly 2-fold (Figure 6B, lanes 1 to 3). These results indicate that Ser 90 phosphorylation may stimulate, and dephosphorylation – inhibit Tat-induced HIV-1 transcription. In a control experiment, equal expression levels were observed for WT CDK9, CDK9 S90A and CDK9 S90D and for co-expressed cyclin T1 (Figure 6C).

### Ser 90 is located on a flexible exposed loop

**Figure 7:**
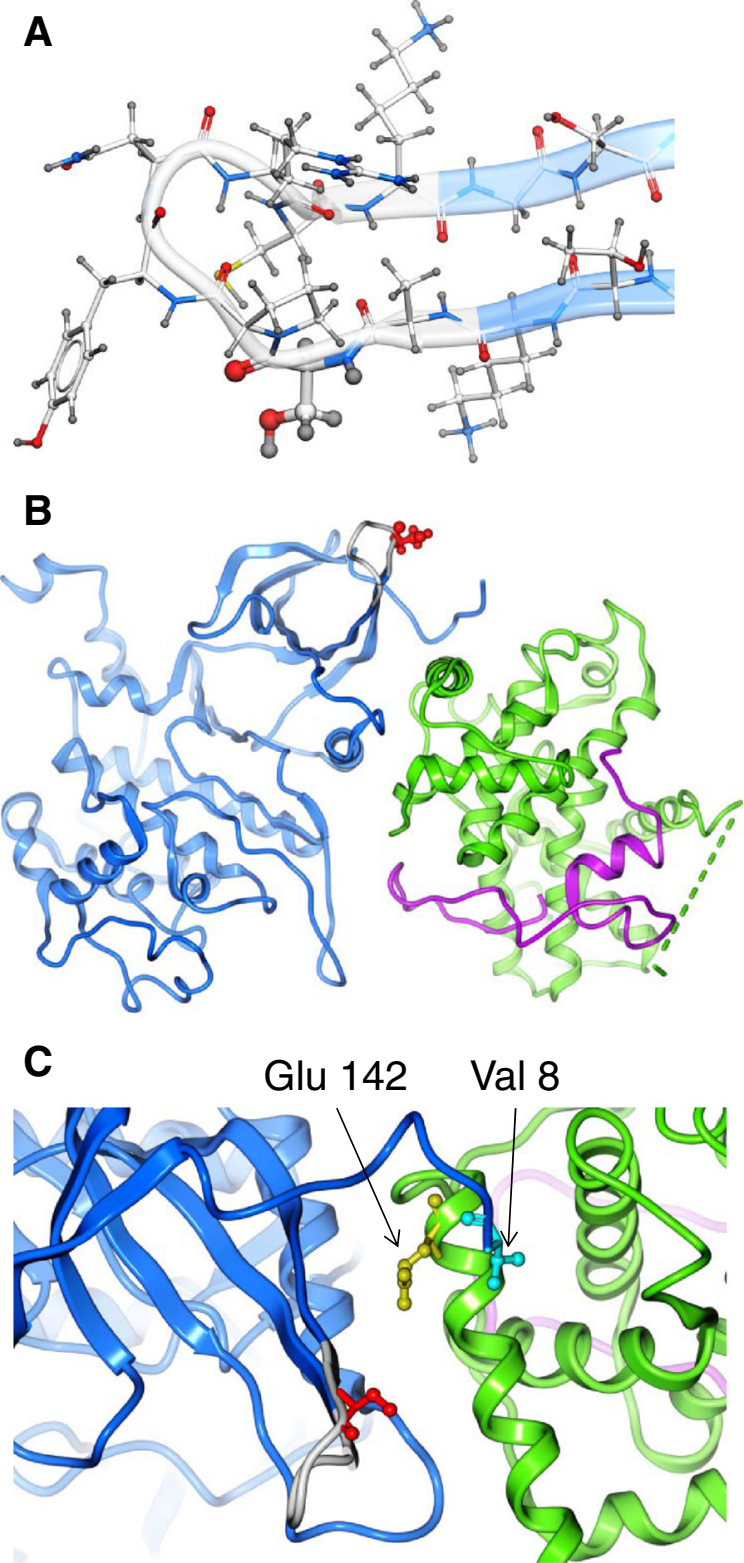
**Modeling of Ser90-containing loop.** (**A)** Structure of Ser90-containing loop of CDK9 (model based on PDB 3MIA). Conformation of residues Thr87-Ser98 with the lowest energy. Backbone representation of residues present in original PDB structure is shown in blue color. Ser90 is emphasized by thickening. **(B)** Structure of CDK9/cyclin T1/Tat complex (model based on PDB 3MIA). Backbone representation of CDK9 residues present in original PDB structure is shown in blue color. CDK9 Ser90 is shown as balls and sticks, in red color. Cyclin T1 and Tat are shown in green and magenta colors respectively. Bullets in cyclin backbone represent missing residues Lys253-Ala260. (**C)** Structure of CDK9/cyclin T1/Tat complex (model based on PDB 3MIA). Backbone representation of CDK9 residues present in original PDB structure is shown in blue color. Cyclin T1 and Tat are shown in green and magenta colors respectively. CDK9 Ser90, CDK9 Val8 and cyclinT1 Gln142 are shown as balls and sticks, in red, cyan and yellow color, respectively. Minimal distance between heavy atoms of CDK9 Ser90 and CDK9 Val8 is 11.38 Å; minimal distance between CDK9 Ser90 and cyclin T1 Gln142 is 12.41 Å. Positions of Val 8 and Gln142 are indicated. Figures were prepared with ICM-Pro software package.

To visualize Ser90 in the CDK9 structure, residues Ala89-Lys96 of CDK9 were modeled with ICM-Pro software package using the coordinates of PDB entry 3MIA [35] as template. Loop sampling resulted in a set of low-energy backbone conformations. The subset of conformations with the lowest energy contains several structures that have expanded beta sheet (residues Leu81-Ala89 and Lys96-Asp104 *versus* Leu81-Thr87 and Ser98-Asp104 in original PDB), while residues Ser90-Cys95 form flexible loop. Local energy minimization of side chains was performed for each of these conformations and the structure with the lowest energy was selected (Figure 7A). The loop was found to be solvent-exposed and to have no specific interactions (Figure 7B). Excluding trivial backbone neighbors, there were no residues within a 10 Å radius of Ser90 (Figure 7C). The absence of residues Ala89-Lys96 in all available X-ray structures and the dissimilarity of several low-energy conformations suggest that this loop is highly mobile.

## Discussion

We have shown here that that CDK2 phosphorylates CDK9 on Ser90 and that this is important for the induction of HIV-1 transcription. Our previous studies showed that HIV-1 transcription is activated by Tat in the G1 phase, but not in the G2 phase [36,37], suggesting that Tat might function in concert with a host cell factor that is expressed in G1. We later found that Tat associates with CDK2/cyclin E [21,22]. Inhibition of CDK2 with siRNA [23] or roscovitin [24] inhibited Tat-induced HIV-1 transcription and HIV-1 replication. Our current study supports these earlier observations, and provides a mechanistic explanation for the effect of CDK2.

We recently showed that iron chelators inhibit CDK2 activity and HIV-1 transcription [28,29]. We also observed that iron chelators inhibit CDK9 activity [28]. The inhibition of CDK9 by iron chelators led to the enticing hypothesis that CDK2 has a direct regulatory effect on CDK9. The present study supports this notion. We show that a siRNA-mediated loss of CDK2 is associated with a decreased CDK9 phosphorylation and activity in cultured cells. CDK9 activity is regulated in part through the association of CDK9/cyclin T1 with 7SK snRNP. In stress-induced cells, the CDK9/cyclin T1 complex dissociates from the large molecular weight P-TEFb complex [7,8]. Similarly, CDK9/cyclin T1 dissociates from the large-molecular weight P-TEFb complex when CDK9 is inhibited with flavopiridol [16]. Interestingly, inhibition of CDK2 resulted in an overall increase in 7SK RNA levels, and the apparent reduction of the large P-TEFb complex. The increase in 7SK RNA could be a compensatory effect to the inability of CDK9 to associate with large P-TEFb complex. Further study is needed to investigate the mechanism of 7SK RNA expression and the effect of CDK2 in this process. The association of CDK9/cyclin T1 with 7SK snRNP requires the phosphorylation of CDK9 on Thr186 [30,31]. Dephosphorylation of CDK9 on Thr186 by protein phosphatase-1 (PP1) disrupts the interaction between CDK9/cyclin T1 and 7SK RNA/HEXIM1 in stress-induced cells [38]. Also, protein phosphatase M1A (PPM1A) was recently shown to dephosphorylate CDK9 on Thr186 in cultured cells [39,40]. We also recently showed that expression of an inhibitory fragment of the PP1 interactor NIPP1 increases the phosphorylation of CDK9 on Thr186, and induces the association of CDK9/cyclin T1 with 7SK RNA, further supporting the notion that PP1 is needed for the dissociation of CDK9/cyclin T1 from the large P-TEFb complex [41]. Thus, it was logical to analyze whether Thr186 phosphorylation was affected in cells with a decreased CDK2 expression. Although we did not detect changes in Thr186 phosphorylation, the overall CDK9 phosphorylation was decreased in the CDK2 knock-down cells, suggesting that different CDK9 residues can be phosphorylated by CDK2. The T-loop of CDK9 contains Ser175, the phosphorylation of which, in addition to Thr186, was shown to affect CDK9 activity [30,31]. Also, phosphorylation of Thr29 was shown to inhibit CDK9 and promote the association of CDK9/cyclin T1 with Brd4 [32].

A CDK2 consensus phosphorylation site adheres to the sequence (S/T)PX(K/R) [34], and is present in CDK9 (S^90^PYNR^94^). Phosphorylation of peptides containing Thr29, Ser175 or Ser90 showed that only Ser90 was phosphorylated by CDK2. These results were confirmed with the CDK9 S90A mutant, which was less phosphorylated by recombinant CDK2 *in vitro* and in cultured cells. Previously, CDK9 was shown to be phosphorylated on at least ten sites that included Thr186 [30,31], Thr29 [32] and the C-terminal residues Ser329, Thr330, Thr333, Ser334, Ser347, Thr350, Ser353, and Thr354 [30]. Thus the residual phosphorylation observed with CDK9 S90A mutant (Figure 4B) can be attributed to one or several of those sites. We recently showed that dephosphorylation of the T-loop Ser 175 residue by protein phosphatase-1 induces CDK9 activity and activates HIV-1 transcription [33]. We also prepared antibodies that recognized phosphorylated Ser90, and found that (i) CDK9 was phosphorylated on Ser90 and (ii) phosphorylation of Ser90 was decreased after the knockdown of CDK2. While we were unable to conclude whether CDK2 KD directly affects formation of the large P-TEFb complex, CDK9 S90A mutant had a decreased association with the large P-TEFb complex. Further analysis is needed to determine whether CDK2 has a direct effect on the formation of 7SK snRNP and whether CDK9 Ser90 phosphorylation is involved in this process. Previously, 7SK RNA knockdown by siRNA led to increased apoptosis but did not change the expression of P-TEFb dependent genes including integrated HIV-1 LTR, suggesting that 7SK RNA has an important cellular function outside of the regulation of cellular or HIV-1 transcription. Consistent with this our notion that Ser90 phosphorylation is important of HIV-1 transcription regulation, CDK9 S90D induced Tat-dependent HIV-1 transcription, whereas CDK9 S90A was inhibitory. In the absence of Tat, both mutants had little effect on HIV-1 transcription. To understand how Ser 90 phosphorylation affects CDK9, we modeled residues 89 to 96, which are not seen in the crystal structure [35]. The modeling showed that residues Ser 90-Cys 95 form a flexible loop that is exposed to solvent and has no specific interactions. One possible explanation for the effect of Ser 90 phosphorylation may be a conformational change that may affect the binding of CDK9 and cyclin T1 to HEXIM1. Future structural and biochemical studies are needed to analyze the effect of Ser90 phosphorylation on CDK9 conformation and binding to HEXIM1.

Earlier studies showed that CDK2 activity is required for the entry into S-phase [42,43] and that the expression of a dominant-negative CDK2 arrests cells in G1 [44,45]. But because mice lacking CDK2 are viable [46,47], CDK2 might be redundant for the cell cycle regulation. In cells that lack CDK2, CDK1 forms functional complexes with cyclin D and cyclin E [48,49]. However, the recent use of chemical genetics by Robert Fisher and colleagues showed a non-redundant, rate-limiting role of CDK2 in restriction point passage and entry into the S-phase [50]. Thus partial knock-down of CDK2 in the present study might have preserved the CDK2 function required for the cell cycle progression and may be used as a strategy for anti-HIV-1 therapeutics. Alternatively, activation of CDK2 can be used as a strategy to facilitate CDK9 Ser90 phosphorylation and induce latent HIV-1 provirus. Recently, the CDK9 inhibitor flavopiridol was shown to induce apoptosis in primary chronic lymphocytic leukemia by targeting CDK9, cyclin T1, AFF3/4 and MLLT1 [51]. Thus, development of CDK2 and CDK9 inhibitors may also be useful for the development of cancer drugs.

## Conclusion

Taken together, our study has identified Ser90 as a novel phosphorylation site of CDK9. The phosphorylation of Ser90 by CDK2 represents a novel mechanism of HIV-1 regulated transcription and provides a new strategy for activation of latent HIV-1 provirus.

## Methods

### Materials

293T cells were purchased from ATCC (Manassas, VA). Histone H1 was purchased from Upstate Cell Signaling Solutions (Charlottesville, VA). Anti-FLAG monoclonal antibodies, protein G and protein A agarose were purchased from Sigma (Atlanta, GA). Recombinant CDK2/cyclin E and CDK9/cyclin T1 were purchased from ProQinase (Freiburg, Germany). Antibodies against CDK9, CDK2 and cyclin T1 were purchased from Santa Cruz Biotechnology (Santa Cruz, CA). Anti-CDK9 phospho-Thr186 polyclonal antibodies were a kind gift from Dr. Qiang Zhou (University of California, Berkeley). Preparation of CDK9 Ser 90 phospho-specific antibodies is described below. Horseradish peroxidase (HRP)-conjugated F(ab)_2_ fragment was purchased from GE Healthcare (Piscataway, NJ). All other inorganic reagents were purchased from Fisher Scientific (Fair Lawn, NJ) or Sigma (St. Louis, MO). Radioactive materials were purchased from Perkin-Elmer (Waltham MA). ARC (4-Amino-6-hydrazino-7-beta-D-Ribofuranosyl-7H-Pyrrolo (2,3-d)-pyrimidine-5-Carboxamide) [52] was a gift from Dr. Andrei L. Gartel (Departments of Medicine, Microbiology and Immunology, University of Illinois at Chicago).

### Plasmids

The HIV-1 genomic vector, pNL4-3.Luc.R^-^E^-^ (Courtesy of Prof. Nathaniel Landau, NYU School of Medicine, New York, NY) was obtained from the NIH AIDS Research and Reference Reagent Program. The Tat expression plasmid was a gift from Dr. Ben Berkhout (University of Amsterdam). WT HIV-1 LTR (−105 to +77) followed by the *luciferase* reporter gene (KB SP WT) was kindly provided by Dr. Manuel López-Cabrera (Unidad de Biología Molecular, Madrid, Spain).

### Transfections

293T cells were seeded in 6 well plates to achieve 50% confluence at the day of transfection. The cells were transfected with indicated plasmids using Lipofectamine and Plus reagents (Life Technologies) following manufacturer’s protocol. The efficiency of transfection was verified using a plasmid encoding green fluorescent protein. The cells were cultured for 48 hrs post-transfection and then analyzed for HIV-1 transcription or phosphorylation of CDK9.

### siRNA transfections

CDK2-directed siRNA (siGENOME SMARTpool reagent M-003236-03-0005) and a control siRNA (D-001206-13-05) were purchased from Dharmacon (Dallas, TX). Control siRNA targets firefly luciferase gene. The siRNAs were transfected at final concentration of 100 nM using Lipofectamin reagent (Invitrogen) according to the manufacturer’s recommendations. The siRNAs were incubated with cells for 2 days before cells were lysed for Western blotting analysis or retransfected.

### Stable CDK2-knock-down cell line

293T cells were transfected with HSH000225-1-LvH1 vectors expressing shRNA that targeted ^399^gcttaaggagctttaaccat^418^ (OS211957), ^919^ccaggagttacttctatgc^937^ (OS211958), ^1010^atggacggagcttgttatc^1028^ (OS211959) and ^49^aggcggcaacattgtttca^67^ (OS211960) sequences of CDK2 (GeneCopoeia, Rockville, MD). Stable clones were selected with 10 μg/ml puromycin. Several clones transfected with OS211959 vector showed significant decrease in CDK2 expression and one of these clones, designated as 293T-CDK KD cells, was used for further studies.

### HIV-1 Transfections

293T and 293T-CDK KD cells were co-transfected with pNL4-3 Luc plasmids and CMV-LacZ expression vector. After 48 hours the cells were collected, washed in PBS, and lysed with Steady Lite Luciferase. The samples luminescence was determined in a Luminoskan Perkin-Elmer). The lysates were then used to measure β-galactosidase activity with ONPG-based assay [53]. Luciferase activity was normalized on the basis of the obtained β-galactosidase activity.

### HIV-1 Tat activated transcription

293T cells were co-transfected with CDK9 WT and mutants and KB SP WT. Cells were also co-transfected with Tat-expressing vector. After 48 hrs the cells were collected, washed in PBS, lysed with Steady Lite Luciferase lysis buffer, and luminescence was determined in a Luminoskan. Luciferase activity was normalized to GFP expression.

### VSVG-HIV-1 Infection

VSVG-HIV-1 was added to 293T and 293T-CDK2 KD cells that were seeded in 24-well plate at ~30 confluence. After 48 hours, the cells were washed in PBS, and lysed with Steady Lite Luciferase buffer. Light emission was analyzed in a Luminoskan. To adjust for the cell number, the MTT assay was performed. Control samples were supplemented with 0.5 mg/ml 3-(4,5-dimethylthiazol-2-yl)-2,5-diphenyltetrazolium bromide (MTT) and incubated for 1 hr at 37°C. Media was removed, formazan crystals were solubilized in dimethyl sulfoxide and absorbance was measured at 654 nm. Luciferase activity was normalized for MTT reading at 654 nm.

### Analysis of CDK2 mRNA expression

Total RNA was extracted from cultured 293T and 293T-CDK KD cells using TRIzol reagent according to the manufacturer’s protocol (Invitrogen Corp.). Total RNA (100 ng) was reverse transcribed to cDNA using Superscript™ RT-PCR kit (Invitrogen, Carlsbad, CA), hexamers and oligo-dT were used as primers. Real-time PCR analysis was conducted on Roche LightCycler 480 detection system (Roche Diagnostics) with SYBR Green. The cDNA was amplified in 45 cycles of denaturation at 95°C for 10 seconds, annealing at 60°C for 10 seconds, and extension at 72°C for 10 seconds and primers for β-Actin, CDK2, Cyclin A and Cyclin E. Primer sequences for β-Actin, forward-AGGCTCAGAGCAAGAGAG, reverse-TACATGGCTGGTGTGTTGA, amplicon size 229; CDK2 forward- TTTGCTGAGATGGTGACTCG, reverse- CTTCATCCAGGGGAGGTACA, amplicon size 196 bp; Cyclin A, forward-GAAACTGCAGCTCGTAGGAA, reverse-ACTTTCAGAAGCAAGTGTTCCA, amplicon size 150bp; Cyclin E, forward –AGCACTTTCTTGAGCAACACC, reverse-CGCCATATACCGGTCAAAGA, amplicon size 161bp. Mean Cp values for β-Actin, CDK2, Cyclin A and cyclin E were determined and expression levels determined using ΔΔCp analysis using β-Actin as reference. Unpaired *t*-test was used to test statistical significance.

### Cell cycle analysis of 293T-CDK KD cells

Approximately 1 million cells were fixed in 70% ethanol at -20°C for 2 hours and stained with Propidium Iodide (10mg/ml) containing RNAse (1mg/ml) for 30 minutes. The data were acquired in BD FACSCalibur (BD Biosciences, Jose, California) and analysis was done using FlowJo software. Unpaired *t*-test was used to test statistical significance.

### Separation of large and small P-TEFb complexes by differential salt extraction

293T cells, 293T –CDK2 KD cells that stably express CDK2 shRNA (OS211959) or 293T cells transiently transfected with a combination of Flag-tagged CDK9 (WT or S90A mutant) and cyclin T1 were cultured in DMEM containing 10% fetal bovine serum. Cells were resuspended in Buffer A (10 mM HEPES (pH 7.9), 10 mM KCl, 10 mM MgCl_2_, 1 mM EDTA, 250 μM sucrose, 1 mM DTT, 0.5% NP-40 and protease inhibitors) added at 500 μl/10^7^ cells. The mixture was incubated on ice for 10 min and centrifuged at 1000 x g for 5 min to pellet the nucleus. The supernatant was removed and saved as the large complex extract (LC). The remaining pellet was resuspended in Buffer B (20 mM HEPES-KOH (pH 7.9), 450 mM NaCl, 1.5 mM MgCl_2_, 0.5mM EDTA, 1 mM DTT and protease inhibitors) added at 500 μl/10^7^ cells. The mixture was incubated on ice for 10 min and centrifuged at 10,000 x g for 1 hr. The supernatant was saved as the small complex extract (SC) (11). The LC and SC were resolved by SDS-PAGE and transferred to a PVDF membrane (Millipore, Allen, TX) for Western blotting. The membrane was probed with anti-CDK9 antibodies.

### Immunoprecipitations

293T cells were lysed in whole cell lysis buffer (50 mM Tris–HCl, pH 7.5, 0.5 M NaCl, 1% NP-40, 0.1% SDS) supplemented with protease cocktail. CDK9 was precipitated as indicated with either anti-CDK9 antibodies or anti-Flag antibodies in the case of overexpression of Flag-CDK9 as we previously described [33]. Briefly, 400 μg of lysate and 800 ng of antibodies combined with 50 μl of 50% slurry of protein A/G agarose were incubated for 2 hrs at 4°C in a TNN Buffer (50 mM Tris–HCl, pH 7.5, 150 mM NaCl and 1% NP-40). The agarose beads were precipitated and washed with TNN buffer, resolved on 10% Tris-Glycine SDS-PAGE, transferred to polyvinylidene fluoride (PVDF) membranes and immunoblotted with appropriate antibodies.

### RT-PCR to detect 7SK RNA

RNA was isolated from complexes that co-immunoprecipitated with anti-CDK9 antibodies or nonspecific IgG as controls using TRIzol reagent according the Invitrogen’s protocol. RNA was also extracted from total cell extract or small and large complex extracts prepared as described above. Total RNA was reverse transcribed using SuperScript II kit (Invitrogen) with the 7SK reverse primer. The following primer sequences were used: forward primer 3^′^-GGATGTGAGGCGATCTGGCTG-5^′^; reverse primer 3^′^-TAAAGAAAGGCAGACTGCCAC-5^′^. Theses primers were used in a PCR reaction that followed the RT reaction. Real-time PCR analysis was conducted on Roche LightCycler 480 detection system (Roche Diagnostics) with SYBR Green. The cDNA was amplified in 45 cycles of denaturation at 95°C for 10 seconds, annealing at 60°C for 10 seconds, and extension at 72°C for 10 seconds. To quantify the amount of 7SK RNA, serial dilutions of 7SK RNA expressing vector [54] was used to determine the copy number. For ΔΔ Cp analysis of 7SK RNA in small and large complex extracts, RNA was reverse transcribed with hexamers and U6 RNA was used as reference. In semi-quantitative PCR, 7SK RNA was amplified for 30 cycles, resolved on 2% agarose gel and photographed.

### CDK9 kinase assay

Kinase assay was performed at 30°C for 30 min in a kinase assay buffer (50 mM HEPES-KOH, pH 7.9, 10 mM MgCl_2_, 6 mM EGTA, 2.5 mM DTT) containing 100 ng of GST-CTD as substrate, 200 μM cold ATP and 5 μCi of (γ-^32^P) ATP. Kinase reactions were stopped with SDS-loading buffer and resolved on 10% PAGE. The dried gel was exposed to Phosphor Imager screen.

### CDK2 phosphorylation assay

293T and 293T-59 cells were lysed in whole cell lysis buffer (50 mM Tris–HCl, pH 7.5, 0.5 M NaCl, 1% NP-40, 0.1% SDS) supplemented with protease cocktail. CDK2 was precipitated with anti-CDK2 antibodies as described above. Kinase assay was performed at 30°C for 20 min in the kinase assay buffer containing 2 μg Histone H1 as substrate, 200 μM cold ATP and 5 μCi of (γ-^32^P) ATP. Kinase reactions were stopped with SDS-loading buffer and resolved on 10% PAGE. The dried gel was exposed to Phosphor Imager screen.

### Phosphorylation of CDK9-derived peptides

We designed synthetic peptides containing CDK9 residues Thr29 (^21^KLAKIGQGTFGEVFK^35^), Ser90 (^88^KASPYNRCKGSIYL^101^); and Ser175 and Thr186 (^172^RAFSLAKNSQPNRYTNRVV^190^). The peptides were phosphorylated by recombinant CDK2/cyclin E in a 10 μl reaction with 100 μM ATP (1μCi of γ-(^32^P) ATP) in the kinase assay buffer using 4 μg of a peptide per reaction for 20 min at 30°C. The reaction was stopped by the addition of 4X SDS-PAGE loading buffer and resolved on a 15% Tris-Tricine gel. The gel was stained with Coomassie blue for 10 min, destained for 1 hr, dried, and exposed to Phosphor Imager screen.

### Constructing CDK9 S90 mutants

QuikChange XL Site-Directed Mutagenesis Kit (Stratagene) was used to generate mutants of CDK9 with the substitutions in the sites of CDK2 phosphorylation determined previously. Primers for S90A substitution were GATTTGTCGAACCAAAGCTGCCCCCTATAACCGCTGC (forward) and GCAGCGGTTATAGGGGGCAGCTTTGGTTCGACAAATC (reverse); and for S90D substitution - GATTTGTCGAACCAAAGCTGACCCCTATAACCGCTGC (forward) and GCAGCGGTTATAGGGGTCAGCTTTGGTTCGACAAATC (reverse). CDK9 S90D was created to mimic phosphorylation. PCR reactions were run for 18 cycles with the extension time of 8 min to allow the synthesis of the whole plasmid sequence. PCR products were digested with *Dpn I* to degrade the original template. The PCR products were transformed into XL-Gold cells. Colonies were then picked, and mini preparations were isolated using High Pure Plasmid Isolation kit (Roche Applied Sciences). The obtained clones were sequenced. CDK9 mutants were expressed in 293T cells, immunoprecipitated with anti-FLAG or anti-Cyclin T1 antibodies and tested for phosphorylation of GST-CTD as described above.

### CDK9 Ser 90 phospho-specific antibodies

CDK9-derived ^86^ RTKASPYNR^94^ peptide without or with Ser 90 phosphorylated was synthesized with an additional Cys residue at the N-terminus for coupling to Keyhole limpet hemocyanin (KLH) or BSA. The KLH-coupled phospho-peptide was injected in the rabbit. The polyclonal serum was double affinity- purified on the BSA-coupled phospho- and non-phosphopeptide linked to CNBr-activated Sepharose-4B (GE Healthcare).

### Modeling

The 8 amino acid residues of CDK9, Ala89-Lys96, that were missing in the CDK9 crystal structure [35], were modeled with ICM-Pro software package [55,56], version 3.6-1i (Molsoft LLC, 2010). First, missing residues were built with the ICM homology modeling tool, using CDK9_HUMAN sequence (Swiss-Prot accession number P50750) and coordinates of PDB entry 3MIA [35] as template. Next, conformation sampling of residues Thr87-Ser98 was performed by ICM ShakeLoop tool using ICM loop database and a set of low-energy backbone conformations was obtained. Finally, local energy minimization of side chains by biased probability Monte Carlo procedure provided conformation with the lowest energy.

## Competing interests

The authors declare that they have no competing interests.

## Authors' contributions

DB, AK, TA, NK, AI, MB (Monique Beullens) and SN conducted experiments, discussed and analyzed data. AVI and MGP conducted modeling and discussed data. DB, PRR, MB (Mathieu Bollen), FK and SN designed the study, discussed the results and wrote the manuscript. SN performed overall design, general control and coordination of the study. All authors read and approved the manuscript.
